# A probiotic *Bifidobacterium pseudocatenulatum* converts hesperidin to bioactive hesperetin and improves metabolic dysfunction in aging mice

**DOI:** 10.1080/19490976.2026.2736902

**Published:** 2026-09-23

**Authors:** Zhao-Qing Shen, Tran Thi Dieu Thuy, Chung-Kuang Lu, Thanh-Sang Nguyen, Chien-Yi Tung, Cheng-Yen Kao, Ting-Fen Tsai

**Affiliations:** a Department of Life Sciences and Institute of Genome Sciences, National Yang Ming Chiao Tung University, Taipei, Taiwan; b Institute of Microbiology and Immunology, National Yang Ming Chiao Tung University, Taipei, Taiwan; c National Research Institute of Chinese Medicine, Ministry of Health and Welfare, Taipei, Taiwan; d PhD Program in Clinical Drug Development of Herbal Medicine, College of Pharmacy, Taipei Medical University, Taipei, Taiwan; e The National Genomics Center for Clinical and Biotechnological Applications, Cancer and Immunology Research Center, National Yang Ming Chiao Tung University, Taipei, Taiwan; f Center for Healthy Longevity and Aging Sciences, National Yang Ming Chiao Tung University, Taipei, Taiwan; g Institute of Molecular and Genomic Medicine, National Health Research Institutes, Miaoli, Taiwan

**Keywords:** CISD2 activator, *Bifidobacterium pseudocatenulatum*, hesperetin, sarcopenia, metabolic dysfunction-associated fatty liver disease (MAFLD), glucose homeostasis, insulin resistance, healthy aging

## Abstract

Dietary flavonoids often require microbial metabolism to generate bioactive metabolites that influence host physiology. Hesperidin, a citrus flavanone glycoside, exhibits limited intestinal absorption and depends on gut microbial biotransformation to yield its active aglycone, hesperetin. Hesperetin activates CDGSH iron-sulfur domain 2 (CISD2), a pro-longevity gene whose expression declines with age, and its pharmacological activation has emerged as a strategy to promote healthy aging. Here, we identified a probiotic strain, *Bifidobacterium pseudocatenulatum* CL256B, that efficiently converts hesperidin to hesperetin via coordinated *α*-L-rhamnosidase and *β*-glucosidase activities, achieving ~90% bioconversion efficiency. In naturally aging mice challenged with a Western diet, six-month treatment with probiotic-derived hesperetin significantly improved age-associated metabolic phenotypes, including reduced body weight and adiposity, increased lean mass, enhanced muscle integrity and grip strength, and attenuation of hepatic steatosis. In addition, glucose tolerance, insulin sensitivity, and whole-body energy metabolism were improved. Transcriptomic analyses revealed activation of SIRT1 and HNF4α signaling in the liver and restoration of ribosome homeostasis in skeletal muscle. These findings support a microbiome-enabled strategy in which probiotic-assisted hesperetin bioconversion activates the CISD2 longevity pathway to improve metabolic health during aging.

## Introduction

Aging is a ubiquitous and irreversible biological process characterized by progressive multi-organ deterioration and functional decline. This systemic dysregulation is a key driver of numerous age-related disorders, including sarcopenia, metabolic dysfunction-associated fatty liver disease (MAFLD), insulin resistance, and disturbances in glucose homeostasis.^1^ ^,^ ^2^ Intriguingly, obesity may further accelerate the aging process. The convergence of global population aging and the obesity epidemic has contributed to the emergence of sarcopenic obesity, a rapidly expanding clinical syndrome defined by the coexistence of reduced skeletal muscle mass and function with excess adiposity. The global prevalence of sarcopenic obesity in adults over 60 years of age is estimated to be approximately 11%, exceeding 28% in certain subpopulations.^3^ Importantly, sarcopenic obesity is associated with significantly increased risks of frailty, impaired mobility, prolonged hospitalization, and cardiovascular disease compared with either sarcopenia or obesity alone. Furthermore, emerging evidence indicates a strong association between sarcopenic obesity and MAFLD.^4-6^ MAFLD, and its progressive form metabolic dysfunction-associated steatohepatitis (MASH), represent the most common chronic liver diseases worldwide, affecting approximately 25-30% of the global population and increasing the risk of hepatocellular carcinoma.^7^ Given the central role of the liver in metabolism, detoxification, and immune regulation, liver aging and MAFLD contribute to multiple systemic disorders, including dementia, cardiometabolic disease, diabetes, and sarcopenia.^8^

Current management strategies for sarcopenic obesity and MAFLD rely predominantly on lifestyle interventions, including dietary modification and physical activity, aimed at reducing body weight, preserving muscle mass, and alleviating hepatic steatosis.^5^ Although resistance exercise can improve muscle strength, physical function, and certain aspects of MAFLD, its impact on body composition is often modest and highly variable in older adults, particularly in individuals with established obesity and metabolic dysfunction. Moreover, adherence to long-term exercise programs remains a major challenge in aging populations. Therefore, there is a critical need to develop novel therapeutic strategies and pharmacological interventions to address these age-associated metabolic disorders.

CDGSH Iron-Sulfur Domain 2 (CISD2) is a recognized pro-longevity gene that regulates healthy lifespan in mammals.^9^ CISD2 is primarily localized to the mitochondrial outer membrane, the endoplasmic reticulum (ER), and the mitochondria-associated ER membranes (MAMs). Functionally, CISD2 plays a critical role in maintaining mitochondrial integrity and regulating intracellular Ca^2+^ homeostasis. Notably, an age-dependent decline in CISD2 expression has been observed across multiple tissues, including the brain, heart, skeletal muscle, skin, and liver, during natural aging in mice.^9^ Loss of CISD2 triggers a pathological cascade characterized by disrupted Ca^2+^ homeostasis, mitochondrial dysfunction, and increased ER stress and oxidative stress. This vicious cycle leads to progressive multi-organ degeneration and dysfunction, ultimately resulting in a systemic premature aging phenotype in mice.^10-17^ Conversely, maintaining high levels of CISD2 expression promotes longevity, delays organ aging, and ameliorates multiple age-associated disorders, including sarcopenia, MAFLD, atrial myopathy, and Alzheimer’s disease.^9^ ^,^ ^17-21^ Together, these findings highlight the pivotal role of CISD2 in longevity regulation and strongly suggest that CISD2 represents a promising molecular target for developing therapeutic strategies to delay aging and treat age-related diseases.

Hesperetin is the aglycone form of hesperidin, a citrus flavonoid abundantly present in the peels of citrus fruits. Numerous studies have demonstrated that hesperetin possesses potent antioxidant, anti-inflammatory, and metabolic regulatory properties, which contribute to its protective effects against cardiovascular disease, metabolic syndrome, and age-related disorders.^21^ Recent studies have further shown that hesperetin improves insulin sensitivity and glucose homeostasis in diabetes and obesity through modulation of insulin receptor (IR)/hosphoinositide 3-kinase (PI3K)/AMP-activated protein kinase (AMPK) signaling and suppression of NF-κB-mediated inflammation, while also inhibiting tumor growth by inducing apoptosis and autophagy via the Akt/mTOR axis. These studies highlight hesperetin as a multi-target bioactive compound with strong potential for the treatment of metabolic disorders and cancer.^22-24^ Previously, we identified hesperetin as a potent activator of CISD2 that significantly upregulates CISD2 expression in multiple tissues, including the heart, skeletal muscle, and skin of aged mice. This pharmacological activation promotes healthy longevity without detectable in vivo toxicity following long-term administration (6-7 months).^21^ ^,^ ^25^ ^,^ ^26^ Importantly, the anti-aging and therapeutic effects of hesperetin are largely CISD2-dependent, as its efficacy is significantly attenuated in the absence of CISD2.^25^ ^,^ ^26^ In addition, hesperetin modulates several longevity-associated regulators, including FOXO3a and SIRT1, thereby suppressing cellular senescence and enhancing metabolic functions.^25^ ^,^ ^27^ ^,^ ^28^ Collectively, these findings establish hesperetin as a proof-of-concept compound and demonstrate that pharmacological activation of CISD2 can exert robust anti-aging effects. Therefore, hesperetin represents a promising candidate for development as a functional food or nutraceutical aimed at delaying aging and promoting healthy longevity.

Although hesperetin exhibits multiple health-promoting properties, its naturally occurring precursor, hesperidin, has limited systemic bioavailability. This limitation is primarily due to the presence of a rutinose disaccharide moiety, which hinders its absorption in the small intestine. Consequently, the health benefits of hesperidin largely depend on microbial biotransformation in the colon, where the gut microbiota converts hesperidin into its more bioactive aglycone form, hesperetin.^21^ ^,^ ^29^ This bioconversion process is mediated by *α*-L-rhamnosidase, which hydrolyzes the rhamnose moiety, followed by *β*-glucosidase, which cleaves the remaining glucose moiety, ultimately releasing free hesperetin.^30^ These enzymes are produced by specific commensal bacteria, particularly members of the genus *Bifidobacterium*, which play a central role in flavonoid metabolism. A previous study demonstrated that *B. pseudocatenulatum* WC0403 can convert approximately 46% of hesperidin to hesperetin within 48 hours.^29^ The resulting hesperetin is released in the colon, absorbed by colonocytes, and subsequently enters systemic circulation to exert its biological effects. In addition to their metabolic capacity, *Bifidobacterium* species are recognized probiotics that promote healthy longevity by enhancing antioxidant defenses, attenuating inflammation, and modulating host metabolism.^31-33^ Notably, a decline in *Bifidobacterium* abundance has been associated with human aging, highlighting their important role in maintaining physiological homeostasis.^32^ ^,^ ^34^

However, although *B. pseudocatenulatum* WC0403 is capable of converting hesperidin to hesperetin, the reported bioconversion efficiency remains relatively low.^29^ Such limited conversion efficiency represents a challenge for the development of functional foods or nutraceutical applications. In addition, the biological effects of *Bifidobacterium*-converted hesperetin on age-associated metabolic disorders remain largely unexplored. Therefore, identifying probiotic strains with superior hesperidin-to-hesperetin bioconversion capacity represents a promising strategy to enhance the bioavailability and therapeutic potential of dietary polyphenols.

In this study, we identified a novel *B. pseudocatenulatum* strain, CL256B (Bp-CL256B), which exhibits exceptionally high conversion efficiency, converting nearly 90% of hesperidin into hesperetin within 24 hours. We further investigated the biological effects of Bp-CL256B converted hesperetin (Bp-Hesperetin) on aging phenotypes and metabolic dysfunction using a Western diet (WD)-induced obese mouse model during naturally aging. By integrating molecular pathology and transcriptomic analyses, we elucidated the mechanisms underlying the beneficial effects of Bp-Hesperetin. Our findings highlight the potential of targeted probiotic-assisted bioconversion as a strategy to enhance flavonoid bioavailability and improve metabolic health during aging.

## Materials and methods

### Fecal donor recruitment and eligibility criteria

Fecal samples were collected from healthy donors after obtaining written informed consent. The study protocol was reviewed and approved by the Institutional Review Board of Taipei Veterans General Hospital (Approval No. 2018-08-010CC). Eligible donors were healthy, non-pregnant adults aged 20-65 with no significant medical history. Individuals were excluded if they had infectious diseases (including HIV, hepatitis B/C, syphilis, or tuberculosis), autoimmune or metabolic diseases, or allergic disorders; gastrointestinal disorders (including irritable bowel syndrome, inflammatory bowel disease, chronic diarrhea, or parasitic infection) within the past three months; travel to regions with a high risk of gastrointestinal infections within the past three months; or use of antibiotics, immunosuppressants, chemotherapy agents, or biologics within the past three months. Additional exclusion criteria included receipt of organ transplantation or blood transfusion within the past six months, high-risk behaviors, a family history of colorectal cancer involving more than two first-degree relatives, alcohol abuse, and chronic neurological, psychiatric, or chronic pain disorders.

### Isolation and identification of *Bifidobacterium* strains

Fecal samples were collected from healthy adult donors using Copan Transystem™ 114C collection tubes (Copan Diagnostics Inc., California, USA.) and transported to the laboratory at room temperature under protocols approved by the Institutional Review Board of Taipei Veterans General Hospital (Approval No. 2018-08-010CC). All samples were processed within 2 hours of collection. Each specimen was dissolved in sterile phosphate-buffered saline (PBS), serially diluted, and plated onto de Man-Rogosa-Sharpe (MRS) agar. Plates were incubated under anaerobic conditions at 37°C for 48 hours. PCR with *Bifidobacterium*-specific primers (BIF-F: 5′-CTCCTGGAAACGGGTGG-3′; BIF-R: 5′-GGTGTTCTTCCCGATATCTACA-3′) was used to screen for putative *Bifidobacterium* colonies.^35^ Candidate isolates were further confirmed by amplification and sequencing of the 16S rRNA gene using universal primers 8F (5′-AGAGTTTGATCCTGGCTCAG-3′) and U1492 (5′-GGTTACCTTGTTACGACTT-3′).^36^ *Bifidobacterium* strains were preserved at −80°C in MRS broth supplemented with 16% (v/v) glycerol until further use.

### Bioconversion of hesperidin into hesperetin by *Bifidobacterium* strains

Hesperidin stock solution (3,280 μM) was freshly prepared in dimethyl sulfoxide (DMSO) prior to use. *B. pseudocatenulatum* strain Bp-CL256B grown on MRS agar plates was inoculated into 5 mL of MRS broth, adjusted to an optical density at 600 nm (OD_600_) of 0.2, and cultured anaerobically at 37°C for 48 hours without shaking. The cultures were then harvested by centrifugation at 4,000 × g for 10 min, and the resulting bacterial pellets were resuspended in 3 mL of MRS broth supplemented with 150 μM hesperidin. Following an additional 24 hours of anaerobic incubation at 37°C without shaking to facilitate microbial biotransformation, the cultures were centrifuged again at 4,000 × g for 10 min. To obtain the Bp-Hesperetin preparation, the supernatant was collected and passed through a 0.45 μm membrane filter to ensure the complete removal of bacterial cells, yielding a cell-free conditioned medium. This conditioned medium, enriched with hesperetin and other bacterial metabolites generated via hesperidin biotransformation, was subsequently lyophilized and incorporated into the Western diet for animal administration. Of note, this preparation contained no exogenously added synthetic hesperetin, serving instead as a standardized postbiotic formulation derived entirely from the controlled microbial metabolism of Bp-CL256B. Prepared samples were stored at −80°C until quantification of hesperetin concentration via UPLC-DAD analysis.

### Genome sequencing, assembly, annotation, and analysis

Genomic DNA (gDNA) of Bp-CL256B was extracted from a 10 mL MRS broth culture incubated anaerobically at 37°C for 48 hours without shaking. DNA extraction was performed using the Presto™ gDNA Bacteria Advanced Kit (Geneaid Biotech, Ltd., Taiwan) following the manufacturer’s protocol for gram-positive bacteria. A total of 2 µg of high-quality genomic DNA (gDNA) was used to construct an SMRTbell library according to the PacBio protocol, and sequencing was performed on the PacBio Sequel II platform to generate HiFi reads. The complete genome of Bp-CL256B was submitted to the National Center for Biotechnology Information (NCBI) database (Accession number NZ_CM167786.1; BioProject PRJNA1397526; BioSample SAMN54433277) and annotated using Prokaryotic Genome Annotation Pipeline.^37^ In addition, Bakta, Rapid Annotations using Subsystems Technology (RAST), and Kyoto Encyclopedia of Genes and Genomes (KEGG) were also employed for genome annotation.^38-41^ The Virulence Factor Database (VFDB)^42^ and the Antibiotic Resistance Gene Database^43^ were used to identify putative virulence factors and antibiotic resistance genes. The genome map of Bp-CL256B was visualized using Proksee.^44^

### Sequence alignment and phylogenetic analysis of *α*-L-rhamnosidase and *β*-glucosidase from *B. pseudocatenulatum* strains

To compare the amino acid sequences of *α*-L-rhamnosidase and *β*-glucosidase among different *B. pseudocatenulatum* strains, including Bp-CL256B, six additional *B. pseudocatenulatum* strains were selected based on the availability of complete genome sequences in the NCBI database. These included Bp-IPLA36007 (GenBank accession GCA_000708005.1), a strain previously reported to exhibit hesperidin-conversion activity,^29^ as well as five representative strains (Bp-LFYP29, Bp-DSM20438, Bp-JCLA3, Bp-YIT11027, and Bp-YIT11956). Homologous protein sequences were identified based on functional annotations and conserved domain analysis. Multiple sequence alignments were performed using MAFFT alignment (version 7.526)^45^ with default parameters. Phylogenetic trees were constructed from the aligned amino acid sequences using MEGA (v11) under the Maximum Likelihood method, with the optimal substitution model selected automatically. Bootstrap analysis was conducted with 1,000 replicates to assess tree robustness, and branch lengths represent amino acid substitutions per site. Phylogenetic relationships and sequence conservation were subsequently examined to evaluate the evolutionary relatedness and potential functional divergence of *α*-L-rhamnosidase and *β*-glucosidase among *B. pseudocatenulatum* strains.

### Mouse models

A diet-induced model of metabolic dysfunction during aging was established using male C57BL/6 mice. Animals were maintained under specific pathogen-free conditions with controlled temperature and humidity, under a 12 hours light-dark cycle. To induce MAFLD and sarcopenic obesity, mice were fed a Western diet (WD; Research Diets D12079B) supplemented with one of the following preparations: (a) bacterial medium (MRS), (b) conditioned medium derived from Bp-CL256B cultures (bacterial control), or (c) conditioned medium generated by microbial biotransformation of hesperidin by Bp-CL256B, containing Bp-Hesperetin (20 mg/kg/day) together with other bacterial-derived metabolites. Bacterial conditioned media were first passed through a 0.45 μm filter, lyophilized, and then blended into the WD prior to feeding. Mice were enrolled at 14 months of age, representing late middle age, and randomly assigned to experimental groups. Treatments were continued for approximately 6 months, after which animals were sacrificed at 20 months of age for subsequent analyzes. All procedures involving animals were approved by the Institutional Animal Care and Use Committee (IACUC; No. 1120205r) of National Yang Ming Chiao Tung University and conducted in accordance with institutional and national regulations. The animal experiments were designed and conducted in compliance with the 3Rs principles (Replacement, Reduction, and Refinement), adhering to the statutory guidelines of the Animal Protection Act of Taiwan.

### Body composition and metabolic phenotyping

Body composition, including fat mass and lean mass, was assessed using nuclear magnetic resonance (NMR)-based analysis (Minispec LF50 TD-NMR). Measurements were performed at designated time points to monitor longitudinal changes. Energy metabolism was evaluated using indirect calorimetry. Oxygen consumption (VO₂), carbon dioxide production (VCO₂), energy expenditure and food intake were measured using a metabolic cage system under controlled environmental conditions (TSE Systems PhenoMaster Next Generation 6528).

### Grip strength measurement

Muscle function was evaluated using a grip strength meter (BIOSEB). Each mouse was allowed to grasp a grid, and peak force was recorded as the animal was gently pulled backward. Multiple measurements were obtained per mouse, and the average value was used for analysis.

### Western blotting

Liver and skeletal muscle tissues were homogenized in RIPA buffer (50 mM Tris-HCl, pH 7.4; 150 mM NaCl; 0.5% sodium deoxycholate; 0.1% SDS; 1% Triton X-100) supplemented with protease (Roche, 04693124001) and phosphatase inhibitors (Roche, 04906845001) using a MagNA Lyser (Roche, Basel, Switzerland). Protein lysates were mixed with SDS sample buffer (50 mM Tris-HCl, pH 6.8; 100 mM dithiothreitol; 2% SDS; 10% glycerol) and heated at 100°C for 15 min. Samples were separated by SDS-PAGE (Bio-Rad, Hercules, CA, USA) and transferred onto PVDF membranes (PerkinElmer, NEF1002001PK). Membranes were blocked with 5% non-fat milk in TBST (25 mM Tris-HCl, pH 7.5; 137 mM NaCl; 2.7 mM KCl; 0.1% Tween-20) for 1 hour at room temperature, followed by overnight incubation (14-16 hours) with primary antibodies at 4°C. After washing, membranes were incubated with HRP-conjugated secondary antibodies for 1 hour at room temperature. Signals were detected using ECL reagents (Thermo Fisher Scientific, 34580). Primary antibodies included CISD2^12^ and GAPDH (Millipore, MAB374), with HRP-linked anti-rabbit (Cytiva, NA934) and anti-mouse (Cytiva, NA931) secondary antibodies.

### Histopathological analysis

Liver and skeletal muscle tissues were harvested, fixed in formalin, and processed for paraffin embedding. Tissue sections were prepared and subjected to hematoxylin and eosin (H&E) as well as Masson’s trichrome staining (Muto Pure Chemicals, Tokyo, Japan). For liver immunohistochemistry (IHC), sections were incubated with an F4/80 antibody (BioLegend, 123101), followed by visualization using the Chemicon IHC Select detection system (Millipore, DAB150). Histological evaluation was performed using a light microscope, and representative images were documented. The extent of muscle degeneration was quantified from stained sections using ImageJ software.

### Serum biochemical analysis

Serum samples were collected for biochemical analysis. Liver injury markers, including ALT and AST levels, were measured using an automated clinical chemistry analyzer (NX-600i biochemical analyzer; Fujifilm, Japan).

### Liver triglyceride, malondialdehyde (MDA), and ROS/RNS measurements

Hepatic triglycerides were extracted by homogenizing liver tissues in a chloroform-methanol mixture (1:2, v/v) following established procedures,^46^ and quantified using a commercial triglyceride assay kit (Sigma-Aldrich, TR0100). Lipid peroxidation was assessed by measuring malondialdehyde (MDA) levels using a thiobarbituric acid reactive substances (TBARS) assay kit (Cell Biolabs, STA-330). In addition, hepatic reactive oxygen and nitrogen species (ROS/RNS) were determined using a fluorescence-based assay kit (Cell Biolabs, STA-347). Optical density and fluorescence signals of 2’,7’-dichlorodihydrofluorescein (DCF) were recorded using an Infinite 200 microplate reader (Tecan Group Ltd.).

### Glucose tolerance and insulin sensitivity tests

Systemic glucose regulation was assessed using glucose tolerance tests (GTT) and insulin tolerance tests (ITT).^13^ For GTT, mice were fasted for 6 hours (09:00-15:00) prior to oral administration of glucose (1.5 mg/g body weight). Blood glucose concentrations were recorded at baseline and at designated time points using a glucometer. For ITT, mice were fasted for 2 hours before receiving an intraperitoneal injection of insulin (0.75 U/kg body weight; Actrapid human regular insulin, Novo Nordisk, Bagsværd, Denmark). Blood glucose levels were subsequently measured over time to evaluate insulin sensitivity.

### RNA extraction and transcriptomic analysis

Total RNA was extracted from tissues, primarily gastrocnemius muscle and liver, using standard protocols. RNA integrity and quality were verified prior to sequencing. For transcriptome profiling, RNA-seq libraries were constructed and analyzed using high-throughput sequencing platforms. Sequencing reads were processed and mapped to the reference genome, followed by differential expression analysis to identify differentially expressed genes (DEGs).

### Bioinformatics and pathway analysis

Principal component analysis (PCA) was performed using MetaboAnalyst v6.0. Differential gene expression was determined from normalized count data using DESeq2 (Wald test) with defined thresholds for fold change and adjusted *p*-values (FDR, false discovery rate). To yield a sufficient number of DEGs for robust downstream pathway analysis, FDR cutoff was set at FDR < 0.05 (*p* < 0.0035) for liver tissue, whereas a relaxed threshold of FDR < 0.2 (*p* < 0.0076) was applied to skeletal muscle. To interpret biological relevance, enrichment analyzes were conducted using Gene Ontology (GO) and Kyoto Encyclopedia of Genes and Genomes (KEGG) databases. Upstream regulatory networks were further explored using Ingenuity Pathway Analysis (IPA) to predict key transcriptional regulators. Activation states were evaluated based on z-scores, calculated from normalized expression values relative to the mean and standard deviation (SD), together with statistical significance.

### Statistical analysis

Data are presented as mean ± SD. Statistical analyzes were carried out using standard analytical software (GraphPad Software, v10.0). For comparisons between two groups, unpaired two-tailed Student’s t-tests were applied. Differences among multiple groups were evaluated by one-way analysis of variance (ANOVA) followed by Bonferroni post hoc testing where appropriate. A *p*-value < 0.05 was considered statistically significant. Sample size determination was framed within the constraints of aged animal availability and guided by the 3Rs ethical principle of animal reduction.

## Results

### Screening and identification of novel *B. pseudocatenulatum* strains with high hesperetin bioconversion efficiency

**Figure 1.**
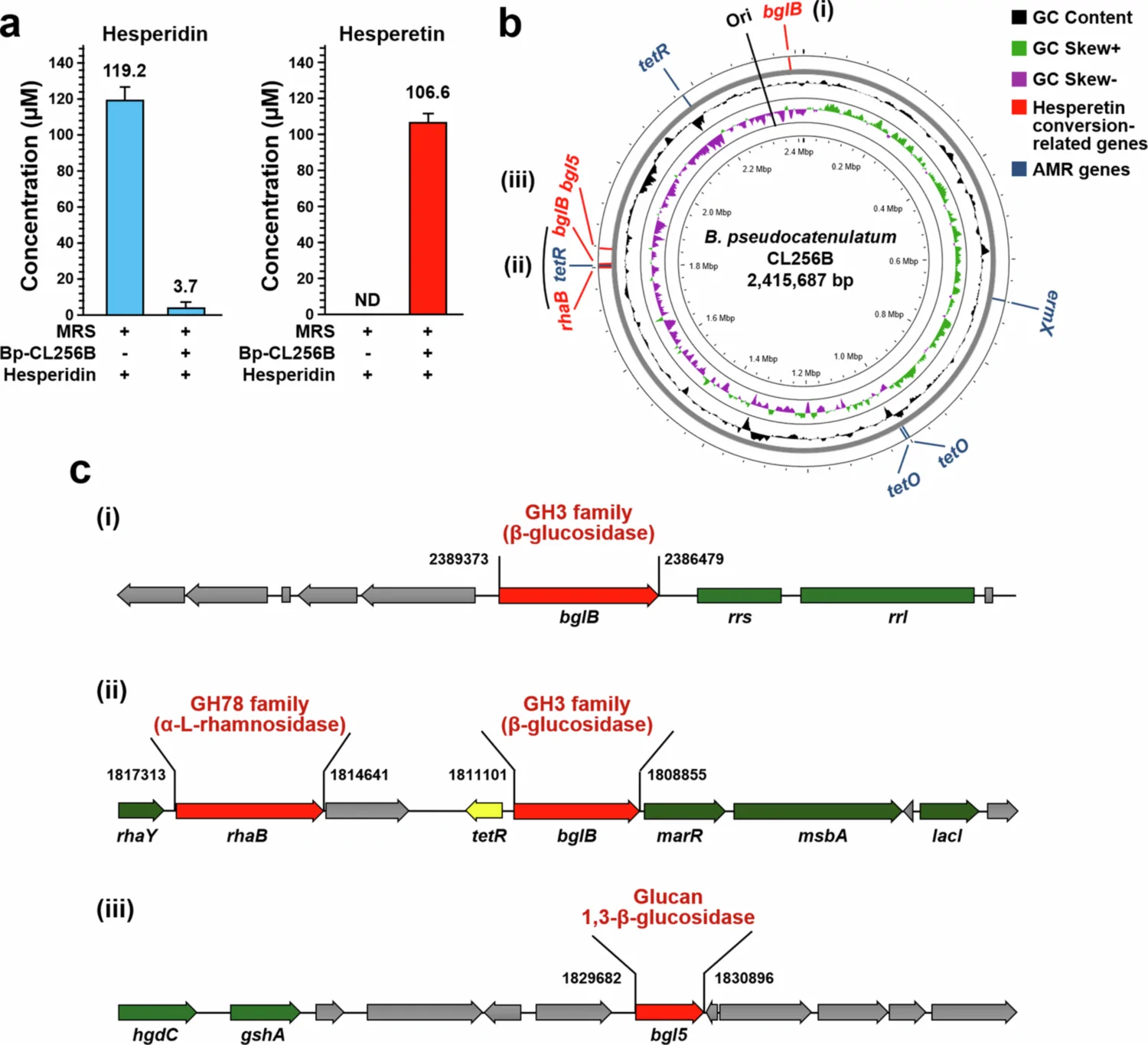
**Bioconversion of hesperetin from hesperidin by** ***B. pseudocatenulatum*** **CL256B (Bp-CL256B) and its whole genome sequencing. (a)** Conversion of hesperidin (blue) to hesperetin (red) by Bp-CL256B was quantified using UPLC-DAD. Hesperidin-MRS broth incubated without Bp-CL256B served as the negative control. ND, not detected. **(b)** A circular genome map of Bp-CL256B showing chromosome size, GC content, GC skew, and targeted annotated coding sequences. Putative hesperidin-metabolizing genes, including *α*-L-rhamnosidase and *β*-glucosidase, are highlighted in red, whereas antibiotic resistance-associated genes are shown in black. The predicted origin of replication (oriC) is indicated. Three genomic regions (i, ii, and iii), defined by their distance from oriC and containing distinct *β*-glucosidase homologs, are highlighted. AMR, antimicrobial resistance. **(c)** Genomic organization of putative hesperidin conversion gene loci. *β*-glucosidase genes are shown in red and *α*-L-rhamnosidase genes in blue to illustrate gene context and clustering. Highlighted regions: region i contains a GH3 family *β*-glucosidase (*bglB*); region ii contains a GH78 family *α*-L-rhamnosidase (*rhaB*) and a GH3 family *β*-glucosidase (*bglB*); and region iii contains a GH5-like *β*-glucosidase gene (*bgl5*). Arrows indicate gene orientation, with arrow length proportional to gene size (bp), and numbers denote the genomic positions of the genes.

To identify *Bifidobacterium* strains capable of efficiently bioconverting hesperidin into hesperetin, fecal samples from healthy adult donors were collected and analyzed. A total of 82 *Bifidobacterium* strains were isolated from 33 fecal samples. Species-level identification revealed substantial diversity among the isolates, with *B. longum* being the most prevalent species (*n* = 56; 68.3%), followed by *B. adolescentis* (*n* = 7; 8.5%), *B. breve* (*n* = 6; 7.3%), *B. pseudocatenulatum* (*n* = 6; 7.3%), *B. faecale* (*n* = 4; 4.9%), *B. bifidum* (*n* = 2; 2.4%), and *B. catenulatum* (*n* = 1; 1.2%). Among the 82 *Bifidobacterium* strains screened for their ability to bioconvert hesperidin, four *B. pseudocatenulatum* strains (designated CYBIF052, CYBIF134, CYBIF136, and CL256B) exhibited detectable conversion of hesperidin to hesperetin after 24 hours of incubation, with conversion efficiencies ranging from 33.4% to 89.4%. Notably, strain Bp-CL256B showed the highest bioconversion efficiency. In this strain, hesperidin levels decreased from 119.2 µM to 3.7 µM, while hesperetin levels increased from undetectable levels to 106.6 µM, corresponding to a conversion efficiency of 89.4% (106.6 µM/119.2 µM) (Figure 1a). Based on this superior conversion activity, Bp-CL256B was selected for further functional characterization and downstream applications.

### Genomic characteristics of *B. pseudocatenulatum* strain CL256B (Bp-CL256B)

To characterize the genomic features underlying the high bioconversion efficiency of Bp-CL256B, we performed whole-genome sequencing (WGS) of this bacterial strain. PacBio sequencing revealed that the Bp-CL256B genome consists of a circular chromosome of 2,415,687 bp with a GC content of 56.6% and no detectable plasmids (**Table S1**). Comparative genomic analysis using BLAST showed that Bp-CL256B shares high sequence identity (98.6%) with *B. pseudocatenulatum* strain YIT11956 (GenBank accession CP079232.1). Functional annotation using RAST and VirulenceFinder detected no genes associated with “Toxins and Superantigens” or “Virulence, Disease, and Defense,” supporting the potential safety of Bp-CL256B for probiotic use.

Two antibiotic resistance genes, *ermX* and *tetO*, along with the regulatory gene *tetR*, were identified in the Bp-CL256B genome (**Table S1;** Figure 1b). The *ermX* gene confers resistance to macrolide, lincosamide, and streptogramin B (MLSB) antibiotics, including lincomycin, clindamycin, erythromycin, quinupristin, pristinamycin, and virginiamycin. The *tetO* gene is associated with resistance to tetracycline-class antibiotics such as tetracycline, doxycycline, and minocycline, while *tetR* functions as a transcriptional regulator of *tetO* expression. Notably, these antibiotic resistance determinants are conserved in several *Bifidobacterium* species, including *B. longum*, an FDA-approved probiotic used in food applications.^47^

Functional annotation further identified several genes potentially involved in hesperetin bioconversion, including three *β*-glucosidases and one *α*-L-rhamnosidase. These *β*-glucosidase genes are distinct in sequence and are distributed across three genomic regions (i-iii) defined by their proximity to the origin of replication (Figure 1b). Region i contains a GH3 *β*-glucosidase (*bglB*), region ii contains a GH78 *α*-L-rhamnosidase (*rhaB*) and a GH3 *β*-glucosidase (*bglB*), and region iii harbors a GH5-like *β*-glucosidase (*bgl5*) (Figure 1c). KEGG annotation classified the *α*-L-rhamnosidase as EC 3.2.1.40, an enzyme involved in flavonoid deglycosylation that is predicted to convert hesperidin into hesperetin-7-O-glucoside. The GH3 *β*-glucosidases in regions i and ii (EC 3.2.1.21) are predicted to hydrolyze glucosylated intermediates into their corresponding aglycones, including hesperetin, naringenin, and isosakuranetin, consistent with the flavonoid profiles detected by LC-MS/MS (**Figure S1**). In contrast, the GH5-like *β*-glucosidase in region iii (EC 3.2.1.58) appears to be primarily associated with general carbohydrate metabolism rather than flavonoid bioconversion.

Consistent with these genomic predictions, substrate specificity assays demonstrated that Bp-CL256B efficiently metabolizes glycosylated flavonoids (**Figure S1a**). Among the tested compounds, hesperidin was converted most efficiently, resulting in substantial accumulation of hesperetin compared with the control (**Figure S1a**). Moderate deglycosylation activity was also observed for didymin and narirutin, producing the corresponding aglycone isosakuranetin from didymin, whereas the expected product from narirutin, naringenin, was not detected. In contrast, negligible conversion was observed for polymethoxylated flavonoids such as tetramethyl-O-scutellarin and tangeretin (**Figure S1b**). Structural comparisons suggest that Bp-CL256B preferentially hydrolyzes rutinoside- or glucoside-containing flavonoids, whereas highly methoxylated or non-glycosylated compounds are poor substrates. Together, these results demonstrate that Bp-CL256B exhibits robust glycosidase-dependent deglycosylation activity with distinct substrate specificity.

### Sequence diversity and evolutionary conservation of *α*-L-rhamnosidase and *β*-glucosidase in *B. pseudocatenulatum* strains

To investigate the evolutionary conservation and sequence diversity of enzymes involved in the bioconversion of hesperidin to hesperetin, we conducted comparative phylogenetic analyzes of glycosidases across multiple *B. pseudocatenulatum* strains. Among the seven strains analyzed, only four strains, namely Bp-IPLA36007, Bp-YIT11956, Bp-YIT11027, and Bp-CL256B, harbored the *α*-L-rhamnosidase gene (**Figure S2a**). Phylogenetic analysis revealed that the *α*-L-rhamnosidase sequences in these strains are highly conserved. Although two distinct clusters were observed, nucleotide divergence among them was minimal (~0.05%). Subsequent amino acid sequence alignment of *α*-L-rhamnosidase using MAFFT identified three variable residues at positions 48, 340, and 852. Notably, compared with Bp-IPLA36007, Bp-CL256B exhibited an aspartic acid-to-alanine substitution at position 852 (**Figure S2b**).

With respect to *β*-glucosidase, all seven strains possessed two GH3 *β*-glucosidases corresponding to regions i and ii in Bp-CL256B, which formed three distinct phylogenetic clusters. Within these clusters, GH3 *β*-glucosidase sequences in region i were more highly conserved than those in region ii (**Figure S2a**). Amino acid sequence alignment revealed that: (i) in region i, the six other strains were highly similar to the region i GH3 *β*-glucosidase of Bp-CL256B, with only six amino acid substitutions at positions 321, 630, 901, 958, 960, and 963. Compared with Bp-IPLA36007, only position 963 (threonine to alanine) differed in Bp-CL256B; (ii) in region ii, more extensive amino acid variation was observed at multiple positions, including residues 4, 9, 65, 159, 212, 276, 294, 313, 328, 364, 365, 367, 396, 567, 569, 575, 577, 596, 618, 620, 621, 633, 645, 669, 671, 679, 689, 693, 700, 705, 741, and 742; and (iii) in region iii, only three strains, namely Bp-CL256B, Bp-YIT11027, and Bp-YIT11956, contained *β*-glucosidase sequences, with only two amino acid differences (positions 226 and 396) among them (**Figure S2b**).

In summary, these results indicate that Bp-CL256B is among the few strains harboring a complete set of hesperetin bioconversion-related genes, including *α*-L-rhamnosidase and *β*-glucosidases. These enzymes contain several unique amino acid residues that distinguish Bp-CL256B from other *B. pseudocatenulatum* strains and may contribute to its enhanced bioconversion capacity.

### Bp-Hesperetin decreases body weight, improves body composition, and ameliorates sarcopenia in naturally aging mice treated with WD

**Figure 2.**
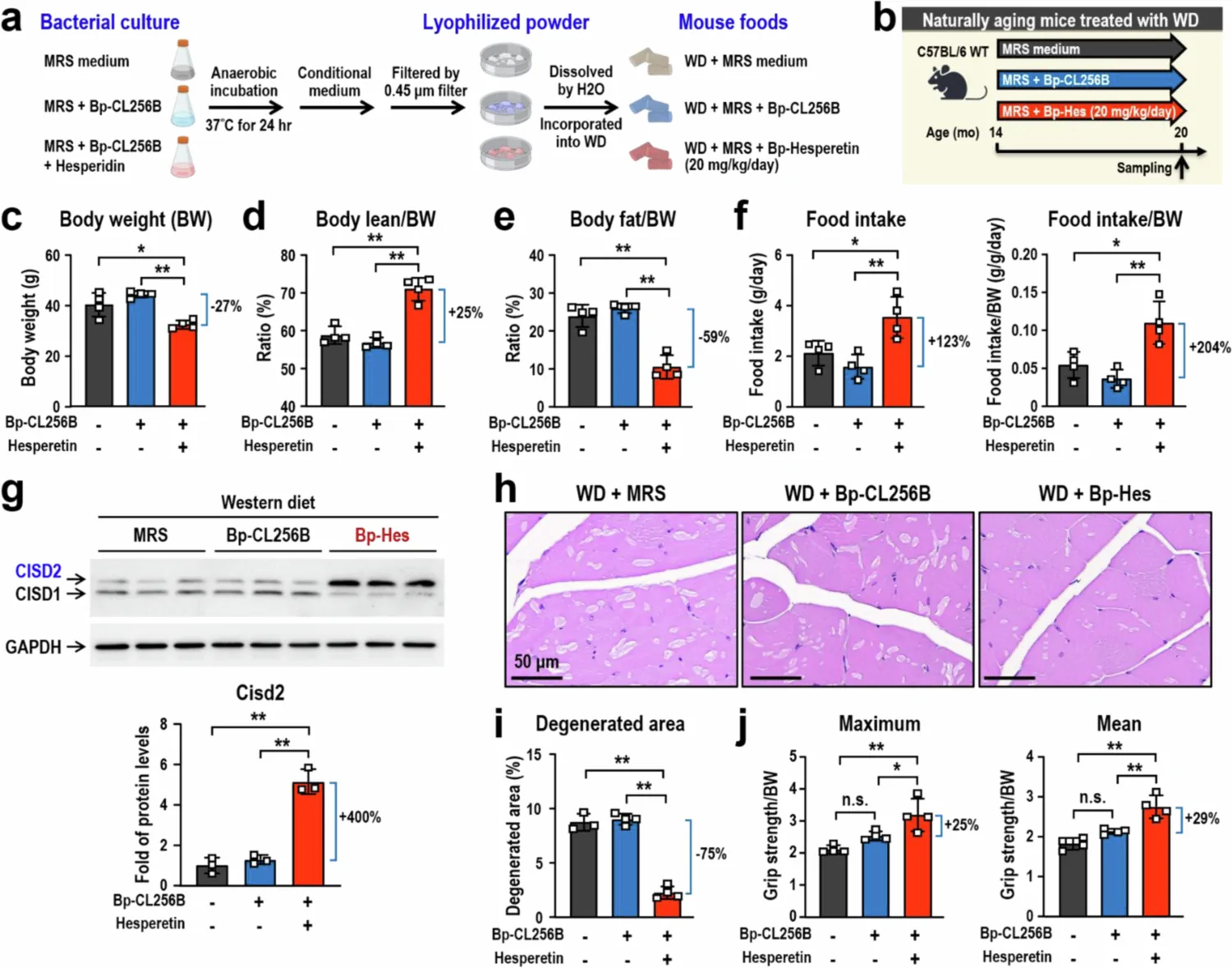
**Bp-Hesperetin activates CISD2, decreases body weight, improves body composition, and ameliorates sarcopenia in WD-treated naturally aging mice. (a)** Schematic workflow for the preparation of WD supplemented with bacterial conditioned medium. Bacterial culture media were harvested after anaerobic incubation at 37 °C for 24 hours and sterile-filtered through a 0.45 μm membrane. The resulting filtrates were lyophilized to produce concentrated powders, which were subsequently dissolved by H_2_O and incorporated into WD. Some illustrations were adapted from BioRender (https://biorender.com/). **(b)** Mouse protocol for investigating the beneficial effects of Bp-Hesperetin on WD-induced and age-related metabolic disorders in middle-aged mice. WT mice at 14-mo old were fed a WD supplemented with conditional medium from (i) MRS (bacterial medium), (ii) MRS with Bp-CL256B (bacteria), or (iii) MRS with Bp-Hesperetin (20 mg/kg/day) for 6 months and sacrificed at 20-mo old. **(c)** Body weight (BW) in different groups of mice (*n* = 4). **(d)** Body lean mass normalized to BW in different groups of mice (*n* = 4). **(e)** Body fat mass normalized to BW in different groups of mice (*n* = 4). **(f)** Food intake in different groups of mice (*n* = 4). **(g)** Western blot analysis and quantification of CISD2 protein levels in the skeletal muscles (gastrocnemius) of different groups of mice (*n* = 3). **(h)** Representative images of H&E staining of skeletal muscle sections prepared from different groups of mice. Scale bar, 50 μm. **(i)** Quantification of degenerated muscle fibers in skeletal muscle of different groups of mice (*n* = 3-4). **(j)** Measurement of grip strength in different groups of mice (*n* = 4). Data are presented as mean ± SD. *p < 0.05; **p < 0.005 by one-way ANOVA with Bonferroni’s multiple comparison test; not significant (n.s.). All the mice used in this study are males.

Previous studies have established that a high-fat, high-sucrose WD effectively recapitulates the clinical features of metabolic syndrome, MAFLD, and sarcopenic obesity in rodent models.^48^ ^,^ ^49^ To investigate the beneficial effects of Bp-CL256B converted hesperetin (Bp-Hesperetin) on WD-induced and age-related metabolic disorders, middle-aged mice (14-month-old) were treated with Bp-Hesperetin (20 mg/kg/day supplemented in the WD) for 6 months (Figure 2a-b). Body composition, molecular pathology of skeletal muscle and liver, and serum biochemical parameters were subsequently evaluated. Body composition analysis, which quantifies whole-body fat and lean mass, was used as an indicator of physical fitness. Strikingly, 6 months of Bp-Hesperetin treatment resulted in a 27% reduction in body weight, a 25% increase in lean mass, and a 59% decrease in fat mass compared with both MRS-treated (medium control) and Bp-CL256B-treated (bacterial control) mice (Figure 2c–e). Intriguingly, although Bp-Hesperetin treatment promoted body weight loss, it concomitantly elevated food consumption in these mice (Figure 2f). Notably, dietary supplementation with Bp-Hesperetin significantly upregulated CISD2 protein levels in the skeletal muscle of WD-treated aged mice (Figure 2g). In addition, Bp-Hesperetin substantially reduced muscle degeneration and significantly improved grip strength compared with the control groups (Figure 2h–j). These findings demonstrate that Bp-Hesperetin effectively reverses sarcopenic obesity and alleviates age-related muscle degeneration and functional decline in WD-treated naturally aging mice.

### Bp-Hesperetin ameliorates MAFLD in WD-treated naturally aging mice

**Figure 3.**
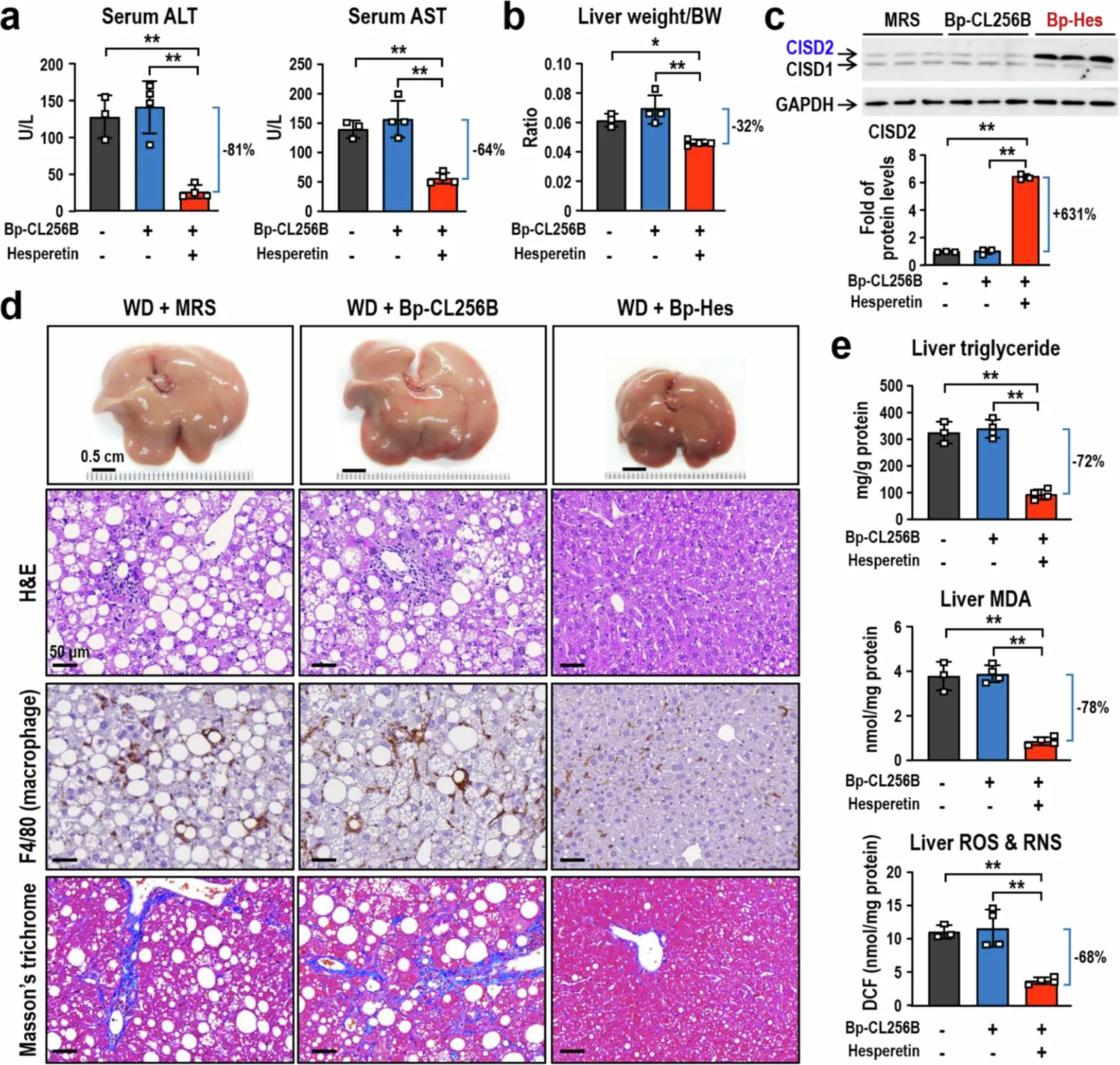
**Bp-Hesperetin activates CISD2 in the liver and ameliorates MAFLD and liver injury in WD-treated naturally aging mice. (a)** Serum ALT and AST levels in different groups of mice (*n* = 3-4). **(b)** Liver to body weight ratio in different groups of mice (*n* = 3-4). **(c)** Western blot analysis and quantification of CISD2 protein levels in the livers of different groups of mice (*n* = 3). **(d)** Liver gross view, H&E staining, IHC staining of F4/80 (macrophage marker) and Masson’s trichrome staining (fibrosis staining) of liver sections prepared from different groups of mice. Scale bar in liver gross view, 0.5 cm. Scale bar in liver section, 50 μm. **(e)** Hepatic levels of triglyceride (TriG), malondialdehyde (MDA, a marker of lipid peroxidation), ROS and RNS in different groups of mice (*n* = 3-4). Data are presented as mean ±SD. *p < 0.05; **p < 0.005 by one-way ANOVA with Bonferroni’s multiple comparison test; not significant (n.s.). All the mice used in this study are males.

WD-induced obesity is a major risk factor for MAFLD, the most prevalent chronic liver disease among older adults worldwide. Serum biochemical analysis of liver injury markers, alanine aminotransferase (ALT) and aspartate aminotransferase (AST), revealed that Bp-Hesperetin treatment significantly attenuated WD-induced liver damage (Figure 3a). Moreover, the liver-to-body weight ratio was markedly reduced by approximately 32% in Bp-Hesperetin treated old mice compared with both the medium control and Bp-CL256B bacterial control groups (Figure 3b). Consistently, dietary supplementation with Bp-Hesperetin significantly upregulated CISD2 levels in the livers of WD-treated old mice (Figure 3c). Histopathological analyzes using H&E staining, IHC, and Masson’s trichrome staining further demonstrated that Bp-Hesperetin effectively alleviated hepatic steatosis, hepatocellular ballooning, liver inflammation, and fibrosis in WD-treated old mice (Figure 3d). In addition, hepatic levels of triglycerides (TriG), MDA, reactive oxygen species (ROS), and reactive nitrogen species (RNS) were significantly reduced following Bp-Hesperetin treatment (Figure 3e). Collectively, these results demonstrate that Bp-Hesperetin effectively ameliorates liver injury and oxidative stress associated with MAFLD in WD-treated naturally aging mice.

### Bp-Hesperetin improves glucose dysregulation, enhances insulin sensitivity, and increases whole-body energy metabolism in WD-treated naturally aging mice

**Figure 4.**
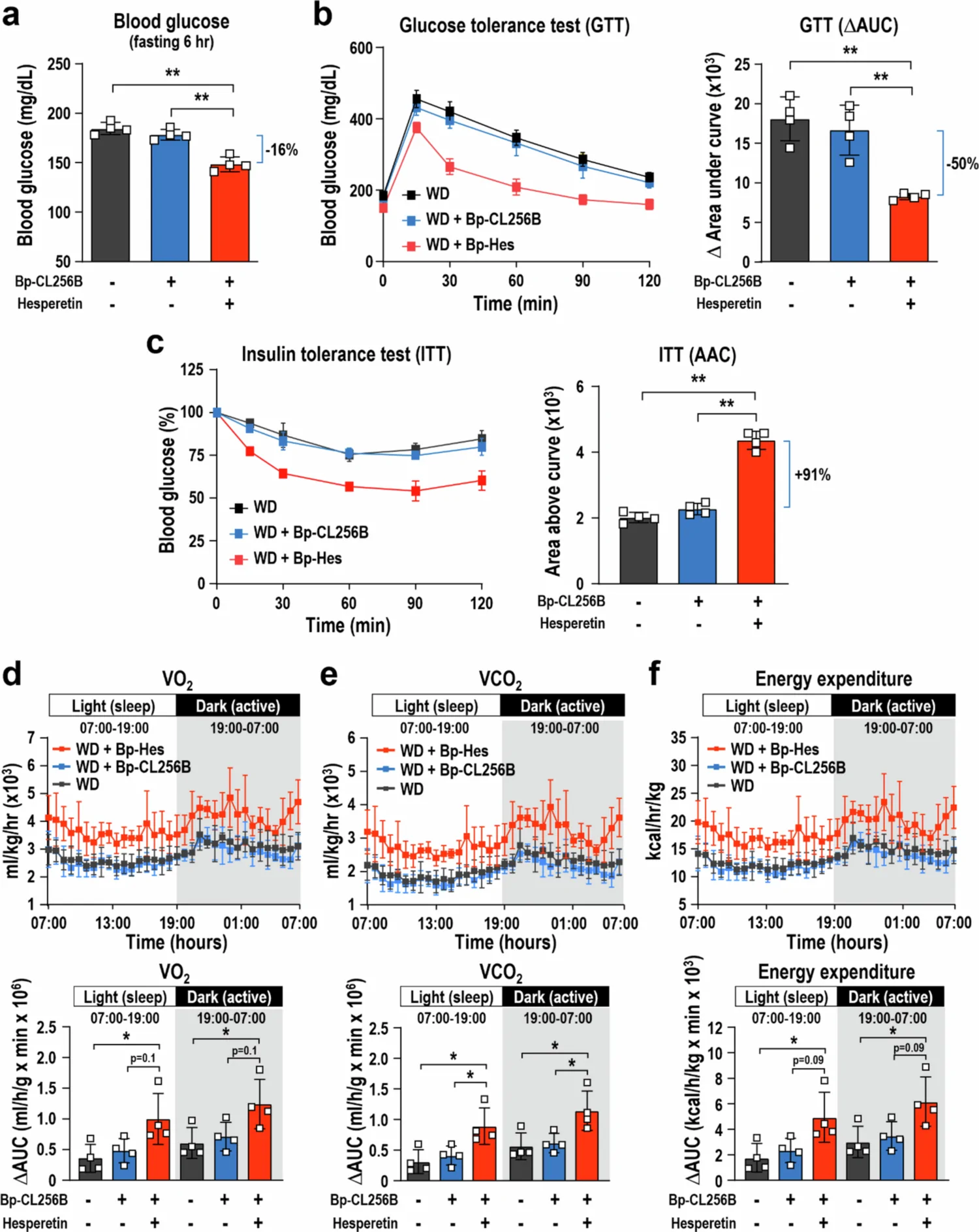
**Bp-Hesperetin alleviates glucose intolerance and enhances insulin sensitivity in WD-treated naturally aging mice. (a)** Basal blood glucose (fasting for 6 hours) in different groups of mice (*n* = 4). **(b)** Oral glucose tolerance test (GTT) (*n* = 4). The quantification of GTT was measured by calculating the delta area under curve (ΔAUC). **(c)** Insulin tolerance test (ITT) (*n* = 4). The quantification of ITT was measured by calculating the area above curve (AAC). **(d-f)** Analysis of whole-body metabolic rate using TSE metabolic cage (*n* = 4). **(d)** Whole-body O_2_ consumption (VO_2_). **(e)** Whole-body CO_2_ production (VCO_2_). **(f)** Energy expenditure. The metabolic rate of individual mice was monitored for 48 hours. The quantification of TSE results was measured by calculating the delta area under curve (∆AUC). Data are presented as mean ± SD. *p < 0.05; **p < 0.005 by one-way ANOVA with Bonferroni multiple comparison test.

Impaired glucose homeostasis, insulin resistance, and disrupted systemic metabolism are hallmarks of WD-associated metabolic disorders. To evaluate the metabolic effects of Bp-Hesperetin on glucose regulation, we performed GTT and ITT. Notably, fasting blood glucose levels (after 6 hours of fasting) were significantly reduced in old mice treated with Bp-Hesperetin (Figure 4a). Furthermore, Bp-Hesperetin treatment significantly improved GTT performance (Figure 4b) and enhanced insulin sensitivity in these mice (Figure 4c). These findings indicate that Bp-Hesperetin improves glucose homeostasis and attenuates insulin resistance in WD-treated naturally aging mice.

In addition, aging is intrinsically associated with metabolic alterations, including a characteristic decline in systemic energy metabolism.^50^ To further assess the metabolic effects of Bp-Hesperetin, we measured whole-body energy metabolism using an indirect calorimetry system (TSE PhenoMaster). Notably, Bp-Hesperetin treatment significantly increased whole-body metabolic activity, as indicated by elevated oxygen consumption (VO₂), carbon dioxide production (VCO₂), and energy expenditure (Figure 4d–f). This elevated whole-body metabolic rate likely accounts for the lean phenotype observed in Bp-Hesperetin-treated mice, despite their higher food intake and caloric consumption (Figure 2c and 2f). Together, these results demonstrate that Bp-Hesperetin counteracts age-associated metabolic decline by enhancing whole-body energy metabolism in WD-treated naturally aging mice.

### Bp-Hesperetin reverses transcriptomic reprogramming associated with MAFLD via activation of SIRT1 and HNF4α signaling

**Figure 5.**
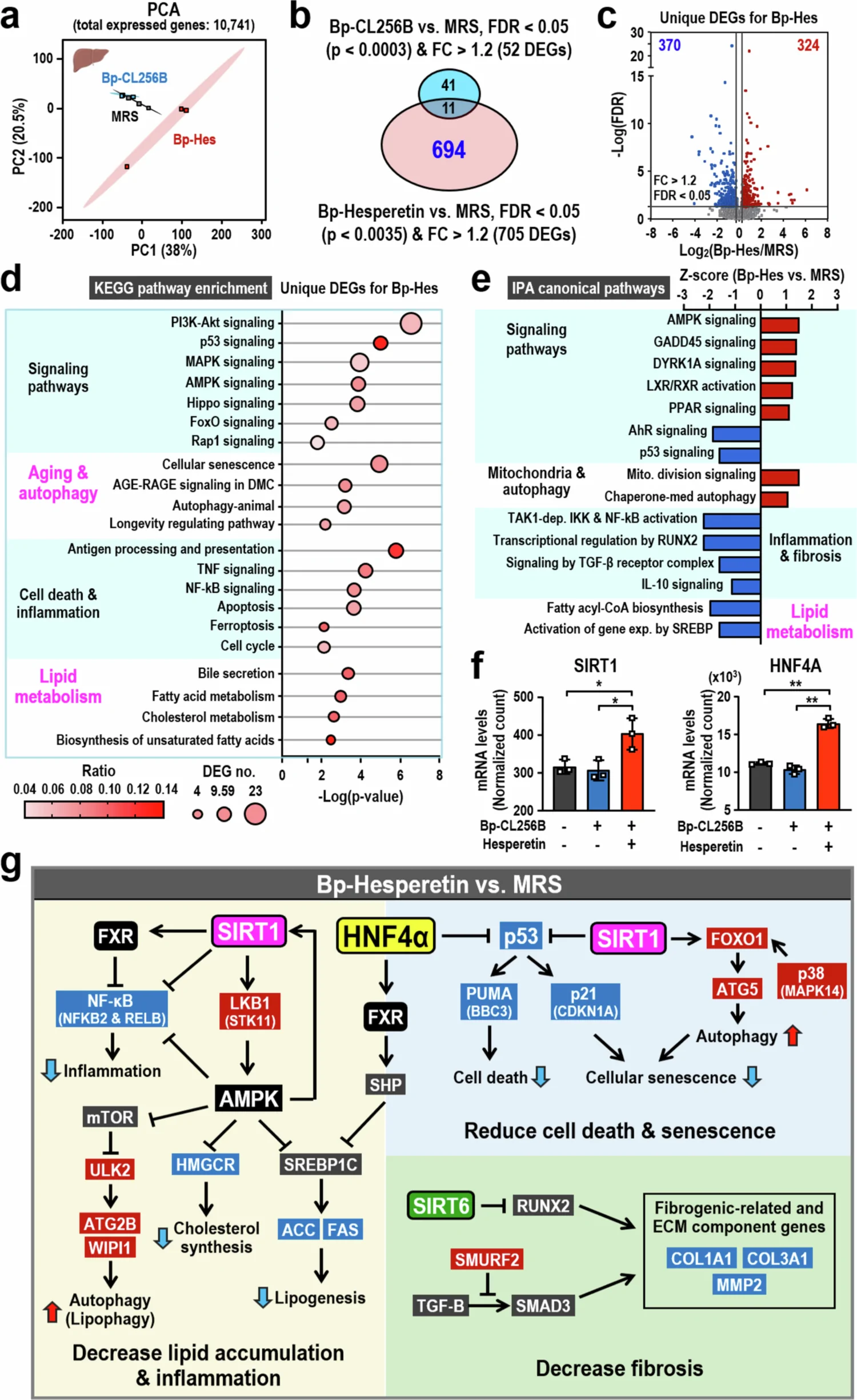
**Bp-Hesperetin drives hepatic transcriptomic reprogramming through activation of SIRT1 and HNF4α signaling. (a)** PCA of liver transcriptomes from different groups of mice after Bp-Hesperetin treatment for 6 months at 20-month-old (10,741 genes; filtered counts > 100 in ≥ 50% samples). **(b)** Venn diagram of DEGs identified by DESeq2 (fold change > 1.2, FDR < 0.05). **(c)** Volcano plot of Bp-Hes specific DEGs (694 genes; 324 upregulated, 370 downregulated). **(d-e)** KEGG and IPA analyzes showing enrichment of pathways related to lipid metabolism, inflammation, cell death, and metabolic regulation (*p* < 0.05). **(f)** The mRNA levels of SIRT1 and HNF4α in the livers of different groups of mice (*n* = 3). The mRNA levels were quantified by RNAseq analysis (*n* = 3; mean ± SD; *p < 0.05, **p < 0.005, one-way ANOVA). **(g)** Schematic summary of pathways regulated by Bp-Hesperetin in the liver. Bp- Hesperetin activates SIRT1 and HNF4α signaling, leading to suppression of lipogenesis (SREBP1c/ACC/FAS), inhibition of inflammation and p53-mediated cell death/senescence, enhancement of autophagy/lipophagy, and attenuation of fibrogenic signaling (TGF-β/SMAD3), collectively contributing to improved hepatic homeostasis.

To investigate the molecular mechanisms underlying the beneficial effects of Bp-Hesperetin on MAFLD, we performed RNA sequencing (RNA-seq) on liver samples from three groups: medium control (WD + MRS), bacterial control (WD + Bp-CL256B), and Bp-Hesperetin (WD + Bp-Hesperetin). A total of 10,741 mRNA transcripts were quantified. Principal component analysis (PCA) revealed that the Bp-Hesperetin group displayed a distinct hepatic transcriptomic profile compared with both control groups, indicating substantial transcriptional reprogramming toward a healthier metabolic state (Figure 5a). Differential expression analysis using DESeq2 (FDR < 0.05; fold change > 1.2) identified 52 differentially expressed genes (DEGs) in the Bp-CL256B group (28 upregulated and 24 downregulated) and 705 DEGs in the Bp-Hesperetin group (332 upregulated and 373 downregulated), indicating a substantially stronger transcriptional response to Bp-Hesperetin (Figure 5b). Intersection analysis identified 694 unique DEGs in the Bp-Hesperetin group, with only 11 DEGs shared between the two groups (Figure 5b-c **; Figure S3a**).

KEGG pathway enrichment and Ingenuity Pathway Analysis (IPA) of these 694 DEGs revealed enrichment in pathways associated with hepatic metabolism and MAFLD pathogenesis, including lipid metabolism, cell death, inflammation, autophagy, aging, and signaling pathways such as p53, AMPK, FoxO, and NF-κB (Figure 5d **; Figure S3b-d**). IPA further classified these pathways into four functional categories: (i) liver metabolism and homeostasis, (ii) mitochondrial function and autophagy, (iii) inflammation and fibrosis, and (iv) lipid metabolism (Figure 5e **; Figure S3b-d**). Notably, metabolic regulatory pathways including AMPK, PPARα, and LXR/RXR were significantly activated in the livers of Bp-Hesperetin treated mice (Figure 5e); activation of these pathways has been shown to ameliorate MAFLD and maintain liver homeostasis.^51-53^ Whereas pathways associated with MAFLD progression and liver aging, such as AhR, p53, NF-κB, RUNX2, and TGF-*β* signaling,^54-58^ were significantly suppressed (Figure 5e).

To identify upstream regulators of these hepatic DEGs, we performed upstream regulator analysis using IPA. A total of 12 significant regulators were identified, including 6 activated and 6 inhibited upstream regulators (*p* < 0.05, absolute Z score > 1.5) (**Table S2**). Notably, SIRT1 and HNF4α, two transcriptional regulators known to suppress MAFLD and promote healthy aging,^59-61^ were identified to be significantly activated and upregulated in the livers of Bp-Hesperetin treated mice (Figure 5f **; Table S2**). Previous studies have shown that SIRT1 maintains hepatic metabolic homeostasis by modulating AMPK, NF-κB, and p53 signaling pathways.^61^ In addition, HNF4α suppresses MAFLD progression by inhibiting p53 signaling and activating FXR, a key regulator of hepatic metabolic homeostasis and anti-inflammatory responses.^62^ ^,^ ^63^ Conversely, two lipogenic transcription factors, SREBP1c and SREBP2, which drive hepatic de novo lipogenesis and cholesterol biosynthesis and contribute to MAFLD pathogenesis,^64^ were predicted to be significantly inhibited. This inhibition was accompanied by downregulation of their downstream target genes involved in lipid and cholesterol synthesis, including ACC, FAS, and HMGCR, in the livers of Bp-Hesperetin treated mice (**Figure S3b; Table S2**).

In summary, these transcriptomic analyzes indicate that Bp-Hesperetin activates SIRT1 and HNF4α signaling while suppressing lipogenic transcriptional programs, thereby modulating key pathways involved in lipid metabolism, inflammation, cell death, and fibrosis. These molecular changes likely contribute to the attenuation of WD-induced and age-associated liver pathology (Figure 5g), and consistent with the phenotypic improvements observed in WD-treated old mice (Figure 3).

### Bp-Hesperetin preserves ribosome homeostasis and protein translation in aging skeletal muscle

**Figure 6.**
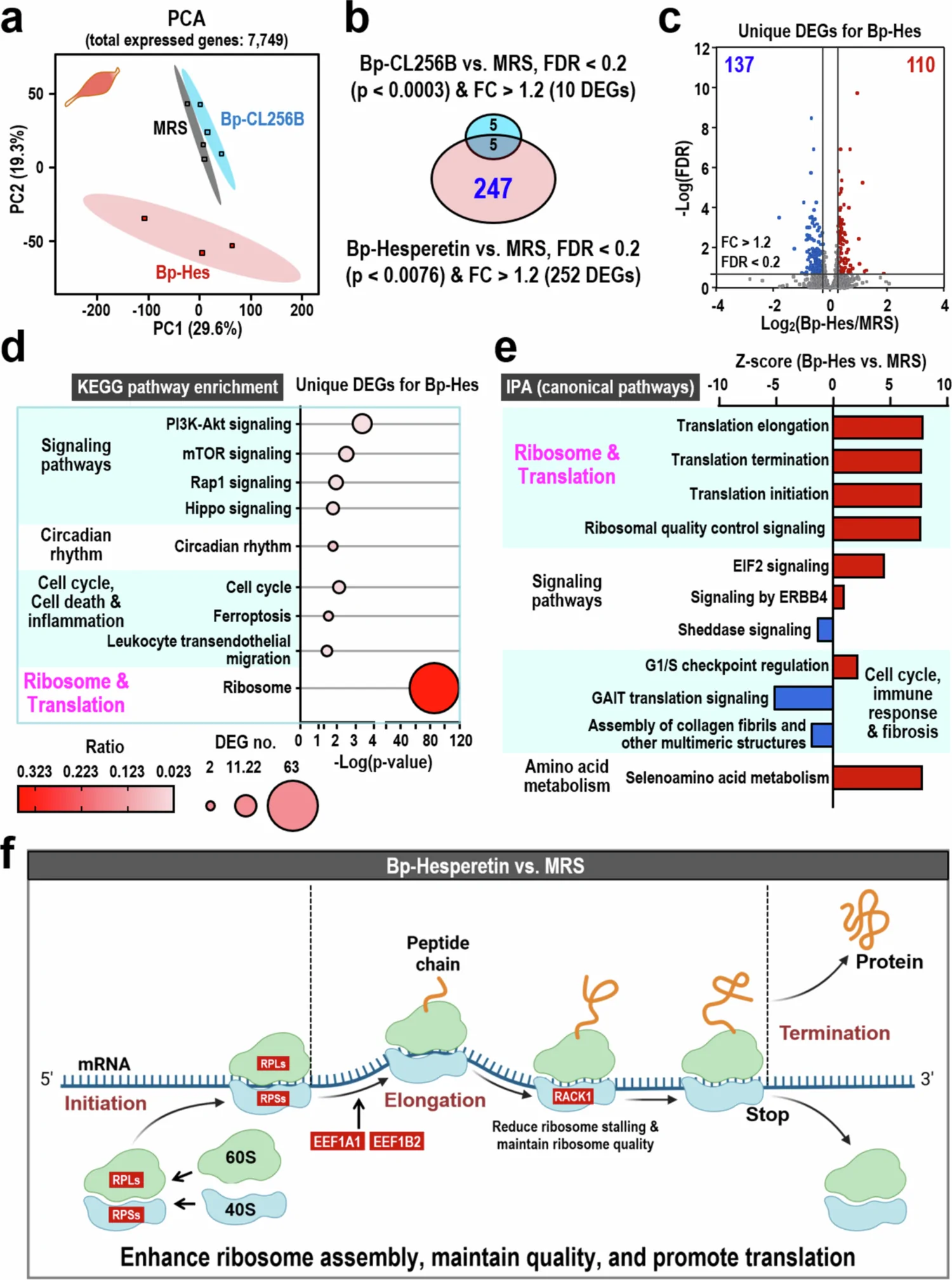
**Bp-Hesperetin restores proteostasis by activating ribosome biogenesis and protein translation in skeletal muscle. (a)** PCA of transcriptomes from gastrocnemius muscle of different groups of mice after Bp-Hesperetin treatment for 6 months at 20-month-old (7,749 genes; counts > 200 in ≥ 50% samples). **(b)** Venn diagram of DEGs identified by DESeq2 (fold change > 1.2, FDR < 0.2). **(c)** Volcano plot of Bp-Hesperetin specific DEGs (247 genes; 110 upregulated, 137 downregulated). **(d-e)** KEGG and IPA analyzes showing enrichment of ribosome- and translation-related pathways (*p* < 0.05). **(f)** Schematic summary of Bp-Hesperetin regulated pathways in muscle. Bp-Hesperetin enhances ribosome biogenesis and translation efficiency by upregulating ribosomal proteins (RPLs/RPSs), elongation factors (EEF1A1, EEF1B2), and the quality control factor RACK1, thereby improving proteostasis in naturally aging skeletal muscle. Some illustrations were adapted from BioRender (https://biorender.com/).

We performed RNA-seq on skeletal muscle from the same three groups. PCA showed that Bp-Hesperetin induced a distinct transcriptomic profile, shifting WD-treated old mice toward a more normal physiological state (Figure 6a). DESeq2 analysis (FDR < 0.2; fold change > 1.2) identified 252 DEGs in the Bp-Hesperetin group (111 up-regulated, 141 down-regulated), substantially more than that in the Bp-CL256B group (10 DEGs), indicating a stronger transcriptional impact. Intersection analysis revealed 247 unique DEGs in the Bp-Hesperetin group (Figure 6b-c **; Figure S4a**). Pathway analyzes using KEGG and IPA consistently showed enrichment of ribosome-related pathways, including components of both large and small subunits, along with activation of translation and ribosomal quality control signaling (Figure 6d-e **; Figure S4b**). Key genes involved in ribosome assembly (RPL10A, RPS19, RPS23, and RPSA), quality control (RACK1, FAU, and UBC), and elongation (EEF1A1 and EEF1B2) were significantly upregulated (**Figure S4b; Table S3**). Given that ribosome homeostasis is critical for muscle mass maintenance and is disrupted during aging, these findings are mechanistically relevant.

In summary, Bp-Hesperetin enhances ribosome biogenesis, preserves translational capacity, and maintains proteostasis in aging muscle, providing a molecular basis for improved muscle mass, integrity, and function (Figure 6f).

## Discussion

This study identifies a novel probiotic strain, Bp-CL256B, with high hesperidin-to-hesperetin bioconversion efficiency, and demonstrates that Bp-Hesperetin exerts multi-organ anti-aging effects in skeletal muscle and liver. Four key findings emerge. **First**, Bp-CL256B achieves ~90% bioconversion efficiency, driven by a conserved repertoire of *α*-L-rhamnosidase and *β*-glucosidases targeting rutinoside-containing flavonoids. **Second**, Bp-Hesperetin upregulates the pro-longevity gene CISD2 and ameliorates WD-induced and age-related phenotypes, including sarcopenic obesity, MAFLD, glucose intolerance, and insulin resistance, accompanied by improved systemic energy metabolism. **Third**, liver transcriptomics reveals modulation of pathways related to lipid metabolism, inflammation, fibrosis, and senescence, with activation of key regulators SIRT1 and HNF4α, supporting improved hepatic health. **Fourth**, in skeletal muscle, Bp-Hesperetin preserves ribosome homeostasis and enhances protein translation, mitigating sarcopenia and maintaining muscle integrity. Taken together, these findings establish a mechanistic and translational framework supporting Bp-Hesperetin as a candidate functional food or therapeutic strategy for targeting muscle and liver aging and related metabolic disorders.

### Bp-CL256B possesses superior capability for hesperidin-to-hesperetin bioconversion

Bp-CL256B exhibits high hesperidin-to-hesperetin bioconversion efficiency compared to the previously reported Bp-IPLA36007, which shows only moderate activity.^29^ This enhanced capacity is likely attributable to increased activity of *α*-L-rhamnosidase and *β*-glucosidases, together with multiple *β*-glucosidases enzyme homologs distributed across distinct genomic loci. The *α*-L-rhamnosidase (EC 3.2.1.40) catalyzes the initial removal of rhamnose from hesperidin and other rutinosides, generating monoglucosides. Subsequent hydrolysis by GH3 *β*-glucosidases (EC 3.2.1.21) yields aglycones such as hesperetin and naringenin, which can undergo further enzymatic modification.^65^ In contrast, the GH5-like *β*-glucosidase identified in region iii (EC 3.2.1.58) appears more closely related to carbohydrate metabolism, indicating that not all *β*-glucosidases in Bp-CL256B directly contribute to flavonoid bioconversion. This functional partition supports the conclusion that GH78 *α*-L-rhamnosidase and GH3 *β*-glucosidases are the principal drivers of flavonoid metabolism in this strain.

Comparative phylogenetic analysis further shows that only a subset of *B. pseudocatenulatum* strains harbor *α*-L-rhamnosidase, providing an explanation for the restricted distribution of hesperidin-to-hesperetin conversion activity observed during screening. Although these enzymes are generally conserved, minor amino acid variations were identified between Bp-CL256B and other strains, including Bp-IPLA36007. While the functional impact of these substitutions remains to be determined, such differences may influence enzyme efficiency or substrate affinity, thereby contributing to the enhanced bioconversion capacity of Bp-CL256B.

From a broader perspective, the strong deglycosylation activity of Bp-CL256B highlights the role of gut microbiota in regulating dietary polyphenol bioavailability. Because many flavonoids are poorly absorbed in their glycosylated forms, microbial conversion into aglycones is essential for host uptake and bioactivity. Bp-CL256B may therefore function as a metabolic “gatekeeper,” enhancing the physiological effects of citrus-derived flavonoids through host-microbe co-metabolism. Taken together, these results demonstrate that Bp-CL256B has high hesperidin-to-hesperetin bioconversion capacity and support its potential application in nutritional and therapeutic strategies targeting metabolic dysfunction, sarcopenia, and other age-associated disorders.

### Bp-Hesperetin exerts hepatoprotective effects

CISD2 expression declines with age across multiple tissues, whereas sustained expression delays aging.^9^ We previously showed that hesperetin is a potent CISD2 activator that promotes healthy longevity and ameliorates age-associated disorders in a CISD2-dependent manner.^25^ ^,^ ^26^ Here, Bp-Hesperetin significantly upregulates CISD2 in the liver and skeletal muscle of WD-treated old mice, supporting probiotic-assisted bioconversion of hesperidin as an effective strategy to generate bioactive hesperetin for functional food or nutraceutical applications.

Upstream analysis identified SIRT1 and HNF4α as key regulators of liver DEGs following Bp-Hesperetin treatment. Both factors were upregulated, consistent with enhanced expression of their downstream targets. Hesperetin has been reported to activate SIRT1, a central regulator of hepatic homeostasis whose activity declines with aging and MAFLD.^28^ ^,^ ^60^ ^,^ ^61^ ^,^ ^66^ Similarly, HNF4α, a master transcriptional regulator of hepatocyte identity and metabolic homeostasis, is reduced during liver aging and MAFLD in both mice and humans.^19^ ^,^ ^59^ ^,^ ^67^ ^,^ ^68^ These findings suggest that the hepatoprotective effects of Bp-Hesperetin are mediated, at least in part, through coordinated activation of SIRT1 and HNF4α.

The mechanistic relationship between activation of SIRT1 and HNF4α and upregulation of CISD2 remains to be defined. Notably, an HNF4α-binding motif is present in the promoter region (−4473 to −4481) of the mouse Cisd2 gene, raising the possibility of direct transcriptional regulation. Future studies should determine whether CISD2 is a direct downstream target of HNF4α and/or SIRT1 in the liver. Taken together, these results highlight CISD2, SIRT1, and HNF4α as interconnected molecular nodes underlying the hepatoprotective and anti-aging effects of Bp-Hesperetin, with translational potential for MAFLD and liver aging.

### Bp-Hesperetin alleviates sarcopenic obesity and preserves muscle function

Ribosome biogenesis is central to cellular homeostasis, regulating proliferation, differentiation, and growth.^69^ In skeletal muscle, tight control of ribosome biogenesis is essential for protein translation and the maintenance of muscle structure and function.^70-72^ During aging, translational efficiency declines, accompanied by disrupted ribosomal stoichiometry, reduced initiation, and increased translational errors, ultimately leading to loss of proteostasis, a hallmark of aging.^73^ ^,^ ^74^ Consistently, aged muscle exhibits reduced ribosome biogenesis and increased translational infidelity.^69^ ^,^ ^75^ ^,^ ^76^ These observations suggest that restoring ribosomal function and translational fidelity may represent a viable strategy to attenuate muscle aging.

Hesperetin has emerged as a candidate modulator of proteostasis, acting through attenuation of ER stress, induction of autophagy, and reduction of protein aggregation.^77-81^ Notably, its pro-autophagic effects have been shown to be CISD2-dependent.^81^ In this study, we identify an additional mechanism: Bp-Hesperetin significantly enhances ribosome biogenesis in aged skeletal muscle (Figure 6d–f; Figure S4b). This is associated with improved ribosomal homeostasis, likely through coordinated regulation of ribosome assembly and translational quality control, thereby mitigating sarcopenia and preserving muscle integrity in WD-treated old mice.

Although the mechanistic link between Bp-Hesperetin and ribosomal regulation requires further investigation, emerging evidence provides a plausible framework. Hesperetin has been reported to activate mTOR signaling in skeletal muscle,^82^ a central regulator of muscle mass and ribosome biogenesis. mTOR drives the activation of key ribosomal components, including RPS6.^70^ ^,^ ^72^ Consistent with this, RPS6 is upregulated in Bp-Hesperetin-treated muscle (Figure S4b), suggesting that activation of the mTOR axis may contribute to the observed enhancement of ribosomal function and proteostasis.

### Limitations and future directions

Several limitations of this study should be acknowledged. **First**, we did not evaluate the ability of Bp-CL256B to colonize the gastrointestinal tract or directly quantify its capacity to mediate the in vivo conversion of orally administered hesperidin. Colonization efficiency is inherently variable among individuals due to host-specific factors, baseline gut microbiota composition, and microbial competition, all of which may influence in situ biotransformation efficiency and therapeutic outcomes.^83^ To minimize these confounding factors and directly evaluate the physiological effects of bacterially generated metabolites, we utilized conditioned medium produced through the microbial biotransformation of hesperidin by Bp-CL256B. This strategy enabled standardized delivery of hesperetin together with other bioactive metabolites, thereby reducing inter-individual variability and providing a more controlled assessment of their therapeutic efficacy. **Second**, although several *Bifidobacterium* species are well-established probiotics, the long-term colonization ability, probiotic characteristics, and safety profile of Bp-CL256B remain to be comprehensively evaluated before clinical translation. Future studies should determine the colonization dynamics of this strain, quantify the in vivo kinetics of hesperidin bioconversion, and assess the therapeutic efficacy of co-administering live Bp-CL256B with hesperidin in both preclinical models and clinical studies. **Third**, direct comparisons among purified hesperetin, co-administration of live Bp-CL256B with hesperidin, and the cell-free Bp-CL256B conditioned medium will be important for defining their individual and potentially synergistic contributions to the observed physiological benefits. Such studies will also help distinguish the effects of hesperetin from those of other bacterial-derived metabolites present in the conditioned medium. **Finally**, several downstream molecular, biochemical, and transcriptomic analyzes were performed using relatively small sample sizes (*n* = 3-4 per group). Such sample sizes are common in long-term aging studies because of the substantial time, cost, and limited availability of aged animals; however, they reduce statistical power. Nevertheless, our conclusions are supported by multiple independent lines of evidence, including improvements in physiological phenotypes, histopathology, biochemical parameters, and transcriptomic profiles. Therefore, the molecular findings should be interpreted with appropriate caution. Future studies using larger cohorts and independent validation will be important to confirm these findings and determine their generalizability across diverse aging populations.

## Conclusions

This study identifies Bp-CL256B as a highly efficient probiotic strain capable of achieving ~90% hesperidin-to-hesperetin bioconversion through specialized *α*-L-rhamnosidase and *β*-glucosidase activities. In naturally aging mice, Bp-Hesperetin robustly ameliorates WD-induced and age-associated phenotypes, including sarcopenic obesity, MAFLD, and glucose dysregulation, by restoring lipid metabolic balance in the liver and preserving ribosomal homeostasis in skeletal muscle, thereby maintaining tissue integrity and function during aging. These results establish a strong mechanistic and translational foundation for Bp-Hesperetin as a next-generation functional food or nutraceutical with significant therapeutic potential.

Importantly, our findings support two complementary and highly promising intervention strategies: (a) direct administration of Bp-Hesperetin as a high-bioavailability bioactive compound, and (b) a synbiotic formulation combining hesperidin with Bp-CL256B to enable in situ bioconversion. Both approaches offer scalable and clinically relevant avenues for promoting healthspan and mitigating age-related disorders. Notably, the effective dose used in this study (20 mg/kg/day) corresponds to a feasible human-equivalent dose (~98.2 mg/60 kg/day^84^) further reinforcing the translational potential of this strategy.

## Supplementary Material

3_BpHesperetin in WD_Supp Tables and Figures_20260914_TF.docx 3_BpHesperetin in WD_Supp Tables and Figures_20260914_TF.docx

## Data Availability

The complete genome sequence of *Bifidobacterium pseudocatenulatum* Bp-CL256B has been deposited in the National Center for Biotechnology Information (NCBI) database under Nucleotide accession number NZ_CM167786.1, BioProject accession number PRJNA1397526, and BioSample accession number SAMN54433277. The data are publicly available at https://www.ncbi.nlm.nih.gov/nuccore/NZ_CM167786.1/, https://www.ncbi.nlm.nih.gov/bioproject/PRJNA1397526 and https://www.ncbi.nlm.nih.gov/biosample/SAMN54433277 Additional data supporting the findings of this study are available from the corresponding author upon reasonable request.
