# Supplementary material for: A probiotic Bifidobacterium pseudocatenulatum converts hesperidin to bioactive hesperetin and improves metabolic dysfunction in aging mice
Source: Gut Microbes. 2026 Sep 23;18(1):2736902. doi: 10.1080/19490976.2026.2736902 (PMC13613915; doi:10.1080/19490976.2026.2736902)
Supplement: Supplementary file 1 [file KGMI_A_2736902_SM6624.docx]

**Figure S1. Bioconversion efficiency and flavonoid profiling of *B. pseudocatenulatum* CL256B (Bp-CL256B) treated with hesperidin.** The four experimental groups included MRS (medium only), MRS+Hesperidin (medium with hesperidin), Bp-CL256B (medium with *B. pseudocatenulatum*), and Bp-CL256B+Hesperidin (medium with *B. pseudocatenulatum* and hesperidin). **(a)** Quantitative analysis of hesperidin consumption and hesperetin production. In the treated group (Bp-CL256B+Hesperidin), hesperidin concentration was reduced from 119.2 µM (MRS+Hesperidin) to 3.7 µM, while the aglycone hesperetin accumulated to 106.6 µM, demonstrating a conversion efficiency of nearly 90%. ND: Not Detected. **(b)** Relative ion intensities of flavonoids detected by LC-MS/MS. The treated group (Bp-CL256B+Hesperidin) is characterized by the unique appearance of hesperetin, diosmetin, and isosakuranetin. The detection of diosmetin and isosakuranetin likely results from the bacterial hydrolysis of minor impurities (diosmin and didymin/naringin) present in the hesperidin source. Other flavonoids, such as tetramethyl-O-scutellarin and tangeritin, remained relatively stable, whereas narirutin levels decreased in the treated group, consistent with hydrolysis. Schematic representation of bacterial hydrolysis. Bp-CL256B utilizes cell-associated glycosyl hydrolases (likely α-L-rhamnosidase and β-glucosidase) to selectively hydrolyze rutinosides (hesperidin, didymin, naringin, and diosmin) to their respective aglycones. Compounds lacking glycosidic linkages, such as the polymethoxyflavones tetramethyl-O-scutellarin and tangeritin, are hardly transformed by the strain.

**Figure S2. Phylogenetic analysis of α-L-rhamnosidase and β-glucosidase amino acid sequences involved in hesperidin conversion of *B. pseudocatenulatum* CL256B (Bp-CL256B) and six other strains.** **(a)** Phylogenetic tree of α-L-rhamnosidase amino acid sequences from four *B. pseudocatenulatum* strains carrying the α-L-rhamnosidase gene. Phylogenetic tree of β-glucosidase amino acid sequences from *B. pseudocatenulatum* strains carrying β-glucosidase. Three genomic regions (i, ii, and iii), defined by their distance from oriC and containing distinct β-glucosidase homologs, are highlighted. Phylogenetic trees were constructed using the maximum likelihood method, and branch support was assessed by bootstrap analysis with 1,000 replicates. The scale bar represents sequence divergence. **(b)** Multiple amino acid sequence alignment of α-L-rhamnosidase and β-glucosidase from *B. pseudocatenulatum* strains. Amino acid sequences from Bp-CL256B, Bp-IPLA36007, and representative *B. pseudocatenulatum* strains were aligned using MAFFT (L-INS-i algorithm) and visualized with Jalview. Only regions exhibiting sequence variation among strains were extracted and shown.

**Figure S3. Bp-Hesperetin modulated DEG expression profiles related to lipid metabolism, cell death, inflammation and fibrosis, aging and autophagy in liver. (a)** Heatmap illustrating that Bp-Hesperetin modulated the expression pattern of a panel of unique DEGs in the livers of WD-fed old mice. **(b)** The heatmap pinpoints the lipid metabolism-related DEGs, including fatty acid metabolism, activation of gene expression by SREBP and cholesterol metabolism, in livers from different groups of mice. **(c)** The heatmap pinpoints pathology-related DEGs, including cell death, inflammation and fibrosis, in livers from different groups of mice. **(d)** The heatmap pinpoints aging-related DEGs, including cellular senescence, AGE-RAGE signaling in diabetes complications (DMC), longevity regulating pathway and autophagy, in livers from different groups of mice.

**Figure S4. Bp-Hesperetin modulated DEG expression profiles related to ribosome and translation processes in skeletal muscle. (a)** Heatmap illustrating that Bp-Hesperetin modulated the expression pattern of a panel of unique DEGs in the skeletal muscle (gastrocnemius) of WD-fed old mice. **(b)** The heatmap pinpoints the ribosome and translation-related DEGs, including ribosome, translation initiation, elongation, and termination, in gastrocnemius muscle from different groups of mice.
